# The Hampstead General Hospital

**Published:** 1907-12-07

**Authors:** 


### THE HAMPSTEAD GENERAL HOSPITAL.

### COMMITTEE'S REPORT REJECTED ON A SHOW OF HANDS.—A POLL DEMANDED.

An extraordinary general meeting of the members of the institute known as the Hampstead General Hospital was held at the hospital, Haverstock Hill, on Monday, December 2, "to receive the report of the Committee of Subscribers appointed at the adjourned meeting of July 16 last, and to consider and vote upon the recommendations contained in such report or any amendment thereto." Mr. E. Bond presided.

#### Committee's Recommendations.

The committee, in the report, recommended as follows :—

1. That all future vacancies on the visiting staff of the hospital be filled by the appointment of consulting physicians and surgeons.
2. That the period of appointment for all present and future members of the staff be fifteen years, to be reckoned from the date of appointment, with sixty as an age limit, provided that as regards the four senior members of the present staff the terms stated in sub-Clause 4 of Clause 8 shall apply.
3. That the number of beds allotted to individual members of the existing staff remain as at present.
4. That the North-West London Hospital be amalgamated with the Hampstead General Hospital upon the terms contained in sub-Clauses i. to v. of Clause 11.
5. That three general physicians and two or three general surgeons, together with such of the following as may be thought necessary—viz. a gynecological physician, an ophthalmic surgeon, a physician to the skin department, a physician to the ear and throat department, and a dental surgeon—be appointed by the council by open competition.
6. That a suitable number of beds be allotted to one or two of such general physicians, to one or two of such general surgeons, and to each of the staff of the special departments.
7. That the out-patient department at Hampstead be discontinued except for the purpose of the treatment of casualties and affections of special organs of the body, and of cases which have already been treated in the wards.

The report was signed by Mr. H. A. Harben (chairman), Mr. Walter Baily, Miss Letitia Clarke, Mr. Frank Debenham, and Mr. Donald McMillan.

Miss Clarke, however, wished it to be recorded that, whilst signing the report and recommendations and agreeing with the detail, she was of the opinion that the interests of the hospital and patients alike would have been better assured by retaining a staff of local medical men with the addition of first-rate consultants.

A memorandum by Sir George Barham was appended, in which he expressed regret that he was unable to append his name to the above recommendations, inasmuch as he differed from his colleagues on the main issue as to the desirability of amalgamating the North-West London Hospital with the Hampstead General Hospital. He did so because he considered it would entirely destroy the local character of the hospital.

#### The Position of the King's Fund.

At the outset the chairman read a letter which had just been received from the King's Fund, stating, *inter alia*, that "We observe that the report of Mr. Harben's committee does not recommend the acceptance of our conditions in so far as they relate both to the proposed shortened period after which the staff of the Hampstead Hospital would consist entirely of consultants, and also to the Hampstead Hospital taking over three members of the staff of the North-West London Hospital. Even were this otherwise, we are now informed that the staff of the North-West London Hospital are not themselves satisfied that the latter proposal sufficiently safeguards their interests. Under these circumstances the King's Fund trusts it will be understood that their only desire is to facilitate a transaction which, it was thought, might be of benefit to the public and at the same time agreeable to the subscribers of the two institutions and fair to their medical staffs and officials. While the King's Fund does not feel justified in promising sums which amount to at least £11,000, in addition to the £5,000 granted last year, unless the conditions which were attached are accepted, they have throughout regarded the proposal merely as an offer. Should the subscribers feel that the work which their own generosity provides will be better served by declining the offer made by the King's Fund, they may rest assured that the King's Fund fully recognise that the subscribers have an undoubted right to decide as they think best. On the other hand, we hope the subscribers will recognise that, although the King's Fund are not always able to make the conditions attached to exceptional offers exactly such as will meet the views of everyone concerned, their desire is always to act fairly to all interests, among which the general necessities of the public have to be taken into consideration."

#### The Discussion and Division.

Mr. H. A. Harben moved the adoption of the report. Speaking first with regard to the letter from the King's Fund, he said the only points at issue between the report of the committee and the King's Fund letter were as to the status of those medical officers who were appointed before 1894 and as to the medical officers from the North-West London Hospital who were to have been taken over by the Hampstead Hospital. When the committee waited upon the sub-committee of the King's Fund which conducted the negotiations, it was stated very clearly that that sub-committee, while holding very strongly to the main points in the original proposal, which had been agreed upon between them and the council, would be prepared to consider any question of detail. The letter would rather lead one to suppose that the King's Fund proposals must either be taken as they were or rejected *in toto*. He hoped, however, that was not quite the position, and that the King's Fund would be amenable to reason upon trifling points. The question of the status of the two medical officers appointed before 1894 might be considered as a matter of such comparative detail that the King's Fund would probably give way on that point. With regard to the appointment of medical officers of the North-West London Hospital, the King's Fund would not give way on that point. They had said they would not, except with the assent of the North-West London medical staff. He had no doubt, however, it would not be impossible to get the assent of the medical staff to the committee's proposal with regard to this matter. The members of the committee approached the whole question with an open mind. Personally, he was considerably impressed with the arguments he heard at the meeting in July against the Council's proposals, and, having heard those arguments reiterated and stated much more fully by all the gentlemen most deeply interested in them, the committee came to the conclusion that, on the whole, they ought not to prevail against the proposals of the Council. They came to the conclusions emboded in the report without reference to the fact that they were also the proposals of the Council of the hospital; but, as they had come to those conclusions independently, he thought the subscribers ought to consider the force of them was strengthened considerably by the fact that they were identical with the conclusions of the Council. Whatever decision the meeting might come to—and he thought that arrived at by the committee was the only one which the subscribers could come to without disturbing very much the administration of the hospital—would be loyally observed by everyone connected with the hospital, inside and outside.

Mr. Frank Debenham, in seconding the motion, said it would have given every member of the committee unfeigned pleasure if they could have seen their way to support the idea of the hospital remaining a Hampstead hospital. Unfortunately, they very soon discovered that that was simply impossible; it was not practicable. The only point on which there was any opening for disagreement was on the amalgamation; and here they had a disagreeable task, inasmuch as they were, he would not say between the devil and the blue sea, but between the King's Fund, which had been a great supporter of the hospital, and the well-known feeling in Hampstead itself. They had done their best as a committee, and he felt convinced that the more the subscribers reflected upon the report the more it would meet with their support. The weak point of the minority report was that Sir George Barham seemed to shrink from the idea of the hospital becoming increasingly useful. (Cries of "No.") If, however, there were an increasing strain upon its usefulness and also upon its funds, he (the speaker) did not think that would frighten the people of Hampstead. If they found the extra usefulness of the hospital involved a larger amount of support, he felt sure Hampstead would rise to the occasion; and they certainly ought not to be frightened by the prospect of having to put their hands into their pockets much more deeply, and make, perhaps, more constant appeals for support than they had done in the past.

Mr. E. Collins, the chairman of the Council, said the committee's report and recommendations had been discussed by them; but it was impossible for them as a body to pass any resolution in reference thereto, inasmuch as the matter was *sub judice*. Personally he thought that although there might be some details in the report with which he could not agree, it was undoubtedly an able report, and the committee had done their work so admirably that he felt he would not be studying the interests of the hospital if he voted against it. If the recommendations were adopted he would be prepared to do everything in his power to bring them into proper effect. He thought also that he could rely upon the other members of the Council to co-operate with him in so doing.

### Amendment Moved and Rejected.

Mr. J. S. Bergheim moved an amendment referring the report back to the committee for further consideration. He felt that the committee had done their very best; but their report was the report of only part of their number. The other part not only did not agree with the report, but were against the very fundamental point, which was whether the hospital should be a Hampstead institution or a public one. As only four members of the committee out of the total of six were really in agreement with the report as it stood, the majority was a very small one, whereas any report should be unanimous. He personally was very much impressed with the arguments against the report which were put forward with great clearness by Sir George Barham. He thought it would be the wish of the subscribers, being patients of the local practitioners, that the views which the latter had expressed should be considered and acted upon. The borough was increasing rapidly, and they wanted a hospital for the borough, and not for other parts of London. There was no reason whatever why they should be saddled with what appeared to be a bankrupt concern simply because their own hospital looked as if it were going to be a prosperous one. If the local practitioners were against the institution, what would become of its funds? One and all would stop their subscriptions if they found that the doctors in whom they had so much confidence were against the hospital. (Cries of "No" and "Yes.") The reason the Hampstead people supported it was because it was their own hospital; if it were made a public institution there was no *raison d'être* for their supporting it. He suggested that it be an instruction to the committee to add three members of the medical profession to their number, and that they be appointed by the local division of the British Medical Association.

Dr. Pidcock seconded the amendment, remarking that, in his opinion, the hospital would be quite as well off without the support of the King's Fund as it would be with the King's Fund and the other hospital joined on to it. It would be better, in fact, for it to remain as it was, supported by local people, than have the support of the King's Fund. The practitioners of Hampstead were not at all in favour of amalgamation and the proposed changes in the medical staff—a large majority of them were not, at any rate. In the past they had only taken a lukewarm interest in the hospital, and in the future they would not even go as far as that, but would be rather against the hospital than for it; and this would mean that a great many subscribers would be lost. The medical staff had made several recommendations by way of deputation and otherwise; but these were almost absolutely ignored. One small concession had been made with regard to the out-patients' department, but that was a very indefinite one; there was nothing to prevent a regular out-patients' department being carried on there. He certainly did not see why the King's Fund should come in and say : "You must take over the North-West London Hospital, which is in distress. You are a wealthy borough, and you must support it." That was what it came to.

Mr. Harben said the committee could not possibly accept the amendment. There was not the least chance of their bringing up a report which was appreciably different from that already submitted, and they certainly could not make the committee unanimous if it were not unanimous. He for one could not possibly undertake to reconsider the matter, and if the amendment were carried he was afraid they would have to appoint another committee.

The Chairman said it would be a shameful waste of time to send the report back; it would involve interminable controversy.

The amendment was negatived by a large majority.

Further Opposition.

Mr. Henry Clark said that a large proportion of the report dealt with the medical staff, and very little of it dealt with the financial aspect. The subscribers were not in possession of the arguments which had weighed with the committee in coming to their conclusions. Why should a hospital which was doing such good work be turned into a gigantic metropolitan hospital? They had already had two metropolitan hospitals in Hampstead, and he wanted to know whether the inhabitants were extremely anxious to have a third. In what way would this excellent institution be benefited by amalgamation with the Kentish Town Hospital, which was not prosperous?

A lady subscriber asked what guarantee there was that the King's Fund would continue its contribution after five years. Was the Hampstead Hospital likely to be left at the end of that time with the extra burden of the Kentish Town Hospital on its hands? She thought there should be a public meeting held at the Town Hall of everyone who had helped the hospital or was identified with it, and that the whole case should be laid before them.

Mr. Donald McMillan had no hesitation in saying that, if he could have done it, he would have gone dead against the King's Fund; but he felt he could not conscientiously do so. It was the duty of the committee to put the interests of the hospital first, and it was in that spirit that the majority of the members of the committee arrived at their conclusions. He wished to point out that the North-West London Hospital had had no difficulty in obtaining funds for the working of the institution; it was only when it became necessary to find £50,000 for rebuilding that it got into difficulties. In further remarks the speaker pointed out that at a conference between a committee of the local medical profession and the medical staffs of the Hampstead and North-West London Hospitals a resolution was passed to the effect that in principle there was no objection to the amalgamation of the two institutions, and that from the public point of view it was desirable. He ventured to say that any committee which considered the matter would come to the conclusion that it was the only thing that could be done in the interests of the poor, for whom the hospitals existed. Regarding the financial position of the Hampstead Hospital, it was calculated that there would be a deficiency at the end of the year of £2,500, and that, with the completion of the new building, they would be £5,000 short of what they required.

Dr. Collingwood Andrews said the report was a great disappointment to a large body of the subscribers. (Hear, hear.) They had thought it would contain something in the nature of a compromise; but as it stood it was a wholesale swallowing of the prescription of the King's Fund. He challenged the production of any favourable opinion regarding the report on the part of medical men in Hampstead. Was it possible that the hospital could be carried on in the teeth of the medical opinion of Hampstead? At a public meeting of the profession held in Hampstead the proposal to change the staff of the hospital was unanimously condemned, and it was impossible to conduct it with any hope of financial success if the medical men were to be ignored.

Mr. Harben, in replying to a question which had been put, said there was no definite guarantee of the King's Fund subscription beyond the five years; but he did not see how they could get a guarantee from a public body like that for an indefinite period. The money in the hands of such a fund was distributed on perfectly fair principles, and if the reasonable requirements of any institution were known the money would be forthcoming. In further remarks the speaker said that no one was satisfied with the existing state of things except the members of the medical staff.

After some further discussion the motion for the adoption of the committee's report was negatived by forty votes to twenty-nine.

A poll having been demanded, the Chairman stated that 739 notices had been sent out and the attendance of those entitled to vote numbered eighty-three. In order to get at the opinion of the general body of subscribers it would be well to issue voting-papers. These would be sent out on Wednesday and be returnable on Saturday; scrutineers would meet on Monday, and the result of their scrutiny would be declared by notices affixed to the doors of the hospital and sent to the Press.

The proceedings then terminated.

